# The clinical application of angiostatic therapy in combination with radiotherapy: past, present, future

**DOI:** 10.1007/s10456-017-9546-9

**Published:** 2017-03-31

**Authors:** Lisanne C. Hamming, Ben J. Slotman, Henk M. W. Verheul, Victor L. Thijssen

**Affiliations:** 1 Department of Medical Oncology, VU University Medical Centre, De Boelelaan 1118, 1081 HV Amsterdam, The Netherlands; 2 Department of Radiation Oncology, VU University Medical Centre, De Boelelaan 1118, 1081 HV Amsterdam, The Netherlands

**Keywords:** Angiogenesis, Radiation, Angiostatic drugs, Clinical trials, Cancer, Combination therapy

## Abstract

Although monotherapy with angiostatic drugs is still far from effective, there is abundant evidence that angiostatic therapy can improve the efficacy of conventional treatments like radiotherapy. This has instigated numerous efforts to optimize and clinically implement the combination of angiostatic drugs with radiation treatment. The results from past and present clinical trials that explored this combination therapy indeed show encouraging results. However, current findings also show that the combination has variable efficacy and is associated with increased toxicity. This indicates that combining radiotherapy with angiostatic drugs not only holds opportunities but also provides several challenges. In the current review, we provide an update of the most recent insights from clinical trials that evaluated the combination of angiostatic drugs with radiation treatment. In addition, we discuss the outstanding questions for future studies in order to improve the clinical benefit of combining angiostatic therapy with radiation therapy.

## Introduction

In 2004, more than 30 years after the proposition that targeting the vascularization of malignant tissues might provide a therapeutic benefit [1], the first angiostatic drug was approved by the FDA, i.e., bevacizumab (Avastin^®^) [2]. Currently, bevacizumab, a monoclonal antibody targeting the vascular endothelial growth factor (VEGF), is FDA approved for the treatment of metastatic colorectal cancer (mCRC), metastatic renal cell cancer (mRCC), non-small cell lung cancer (NSCLC) and glioblastoma, while its treatment efficacy in other malignancies is still being investigated. In addition, in the last decade the FDA has approved several other angiostatic drugs for the treatment of different malignancies (Table 1). Despite the increasing number of angiostatic drugs targeting different angiogenic pathways [3–5], thus far only limited clinical benefit of angiostatic therapy has been demonstrated. For example, bevacizumab monotherapy in patients with previously treated mCRC resulted in an inferior overall survival (OS) of 10.2 months compared to a OS of 10.8 months with standard chemotherapy (FOLFOX4) [6], whereas the first-line therapy with sorafenib in patients with mRCC results in a similar progression-free survival as treatment with interferon alpha-2a [7]. A well-known exception to this is sunitinib which has been shown to improve OS in the first-line treatment of patients with metastatic RCC as compared to interferon alpha [8, 9].

**Table 1:** FDA-approved angiostatic drugs for cancer treatment

Drug (trade name)	Main target(s)	Cancer type^a^
Antibodies		
Aflibercept (Zaltrap^®^)	VEGF/PlGF	mCRC
Bevacizumab (Avastin^®^)	VEGF	mCRC, NSCLC, mRCC, Glioblastoma
Ramucirumab (Cyramza^®^)	VEGFR2	Advanced stomach cancer or gastroesophageal junction adenocarcinoma
Panitumumab (Vectibix^®^ **)**	EGFR	mCRC (wt KRAS)
Cetuximab (Erbitux^®^)^b^	EGFR	mCRC (wtKRAS), metastatic non-small cell lung cancer and head and neck cancer
Small molecules		
Axitinib (Inlyta^®^)	VEGF-R1/2/3, PDGFR, c-KIT	RCC
Everolimus (Afinitor^®^)	FKBP12/mTORC1	RCC, breast cancer, NET
Erlotinib (Tarceva^®^)	EGFR	NSCLC, PC
Pazopanib (Votrient^®^)	VEGFR-1/2/3, PDGFR-α/β, c-Kit	RCC, STS
Regorafenib (Stivarga^®^)	VEGFR-2, TIE-2	mCRC, GIST
Sorafenib (Nexavar^®^)	C-RAF, B-RAF, VEGFR-2/3, PDGFR-β,	RCC, HCC, thyroid cancer
Sunitinib (Sutent^®^)	VEGFR-1/2/3, PDGFR-α/β, c-Kit	mRCC, imatinib-resistant GIST, progressive NET in the pancreas
Thalidomide (Thalomid^®^)	Cereblon, unknown	Multiple myeloma
Vandetanib (Caprelsa^®^)	VEGFR-1/2/3, EGFR, RET	Medullary thyroid cancer
Cabozantinib (Cabometyx^®^)	VEGF-R2, c-MET	Advanced RCC, medullary thyroid cancer
Lenvatinib (Lenvima^®^)	VEGF-R1/2/3	Advanced RCC, thyroid cancer

^a^ *m* metastatic, *CRC* colorectal cancer, *NSCLC* non-small cell lung cancer, *RCC* renal cell cancer, *PC* pancreatic cancer, *NET* neuroendocrine tumors, *STS* soft tissue sarcoma, *HCC* liver cancer, *GIST* gastrointestinal cancer

^b^Was also approved by the FDA in 2004 for the treatment of mCRC [2]

Despite their limited benefit as monotherapeutics, both clinical and preclinical studies have shown that angiostatic drugs can improve the treatment efficacy when combined with other treatments, including chemotherapy [10–14], photodynamic therapy [15–17], immunotherapy [18, 19], miRNA-based therapy [4] and radiotherapy [13, 20–23]. Regarding the latter, promising preclinical observations instigated numerous clinical trials exploring the benefit of combining angiostatic drugs with radiotherapy. Five years ago, we evaluated the clinical opportunities and challenges that accompany the combination of radiotherapy with angiostatic therapy [24]. At that time, over 75 trials were still ongoing. Here, we present an updated overview of the outcome of these clinical trials. In addition, we evaluate the novel insights from these studies and discuss the outstanding questions that new trials should answer in order to improve the clinical benefit of combining radiotherapy with angiostatic treatment.

## The rationale behind combining angiostatic drugs with radiotherapy

At the first sight, the rationale to combine radiotherapy with angiostatic drugs appears counterintuitive since the effect of radiotherapy relies on the presence of oxygen [25] while angiostatic drugs aim to block tumor oxygenation. Despite this apparent conflict, several preclinical studies have shown that angiostatic treatment can enhance tumor oxygenation, thereby increasing the efficacy of radiation treatment [13, 22, 26–28]. The mechanisms by which angiostatic drugs improve tumor oxygenation are still not fully understood. Initially, it was hypothesized that selective killing of the endothelial cells would reduce their oxygen consumption and increase vascular permeability. This would result in an increased oxygen availability and diffusion into the tumor tissue [29, 30]. Later studies indicated that angiostatic treatment might improve tumor oxygenation by remodeling of the abnormal and dysfunctional tumor vasculature to a more normal and functional phenotype [27, 31]. This ‘vascular normalization’ is hypothesized to result from restoring the balance between pro- and anti-angiogenic signals. It results in more stable vessels, lower interstitial fluid pressure, better perfusion and consequently a better overall tumor oxygenation [22, 32–34]. For example, our previous work has focussed on the role of galectins in tumor angiogenesis and cancer [35–40]. This was instigated by our discovery of galectin-1 as a pro-angiogenic factor that is essential for endothelial cell function during tumor angiogenesis [41–44]. Importantly, we identified galectin-1 as the endothelial cell target of a synthetic angiostatic peptide named anginex [41, 45]. Treatment of murine tumor models with anginex (or bevacizumab) was shown to improve tumor oxygenation and consequently to enhance the anti-tumor effect of radiotherapy [22]. This is in line with other preclinical studies which have linked vascular normalization to an enhanced efficacy of radiation treatment [13, 27]. At the same time, it has been shown that the vascular normalization occurs only transiently and that continuation of angiostatic treatment eventually causes vessel regression and reduced tumor oxygenation [22, 31, 34]. Hence, adequate scheduling is important to ensure that radiation is applied during the normalization window [22]. In addition, to what extent vascular normalization occurs in the clinical setting and whether or not it contributes to better tumor oxygenation is still under debate [46, 47]. While the latter still requires further investigation, the potential effects on tumor oxygenation certainly provide a rationale to combine angiostatic drugs with radiotherapy.

Another rationale to combine angiostatic therapy with radiotherapy is the observation that tumor irradiation can directly affect tumor vascularization, perfusion and oxygenation. Such radiation-induced vascular changes appear to be dependent on the dose-scheduling regime. Based on a literature study, different dose-dependent effects of radiotherapy on the vasculature could be distinguished, i.e., vessel deterioration, vessel preservation and vessel induction [48]. The latter, i.e., the stimulation of tumor vascularization and perfusion, is predominantly observed during fractionated (low-dose) radiotherapy. For example, Mayr et al. [49] used contrast enhanced MRI to determine tumor perfusion in cervical cancer patients receiving fractionated radiotherapy (±5 × 2 Gy/week for 4–5 weeks). They observed increased perfusion after 2 weeks of treatment after which a decline in perfusion was observed. A comparable finding was reported by Shibuya et al. [50] using perfusion CT. Improved tumor perfusion following fractionated irradiation was also reported in other tumor types, including in non-locally advanced rectal tumors (5 × 5 Gy) [51], and inoperable non-small cell lung tumors (6 × 4.5 Gy) [52]. In the latter study, the increase in tumor blood volume occurred both at the rim and the center of the tumor albeit to a lesser extent in the tumor center [52]. We also observed improved perfusion in the center of xenograft colorectal tumors in mice that received 3 weeks of fractionated irradiation (5 × 2 Gy/week) [28]. Interestingly, the observation that fractionated irradiation can induce tumor tissue perfusion is in line with reports that fractionated radiotherapy can improve tumor oxygenation [53, 54]. Thus, improved tumor perfusion might represent an additional mechanism of radiotherapy-induced tumor oxygenation, next to previously described mechanisms like decreased oxygen consumption, increased inflammation and reduced tumor volume [55]. While the improved perfusion and oxygenation might increase the efficacy of subsequent irradiations, our recent findings confirm that the tumor areas with increased perfusion also contained more viable tumor tissue [28]. Apparently, the improved tumor vascularization and oxygenation can also contribute to tumor cell survival or to tumor regrowth following fractionated radiotherapy. Thus, blocking this effect by angiostatic drugs could improve the efficacy of the radiation treatment.

Altogether, the addition of angiostatic drugs to radiotherapy might be effective by (1) transiently improving tumor oxygenation and/or (2) counteracting radiotherapy-induced tumor (re)vascularization to prevent or delay tumor recurrence. However, it is evident that optimal dose-scheduling of both treatment modalities is the key to achieve beneficial effects of the combination therapy. After all, dose-scheduling of angiostatic drugs will influence whether and when vessel normalization occurs, thereby affecting the efficacy of radiotherapy. At the same time, dose-scheduling of radiotherapy will influence tumor perfusion and oxygenation, thereby affecting the efficacy of angiostatic drugs. All this has been recognized and studied by us and others in different preclinical tumor models [22, 23, 56, 57]. The current challenge is to translate all these insights into clinically applicable protocols. For this, several outstanding questions have to be answered, especially with regard to dose-scheduling and with regard to the commonality of the observed effects of radiotherapy on tumor vascularization and perfusion. Insights from past, present and future clinical trials on the efficacy of the combination therapy can help to answer these questions.

## The past results of combined angiostatic/radiation therapy

In 2012, we performed an extensive review of the results of clinical trials that combined radiotherapy with angiostatic treatment. The overall conclusion at that time was that this combination treatment generally results in favorable outcomes with regard to tumor response and patients survival [24]. The observed efficacy appeared to depend not only on the type of drug and the type of tumor but also on the proper scheduling and dosing of both therapies which was in line with preclinical observations. In fact, exploring the optimization of dose-scheduling was identified as an important future challenge, especially since concerns were raised regarding the increased toxicity that is observed in patients who received the combination treatment [24, 58, 59]. Interestingly, our recent studies in preclinical tumor models indeed show that optimizing the treatment schedule does allow dose reductions without loss of treatment efficacy, i.e., decrease in tumor volume [23, 28]. This is in line with a previously published mouse study [60] as well as with several clinical case reports [61, 62]. However, strong clinical evidence that dose reduction does result in lower toxicity while not affecting the efficacy of the combination treatment is still lacking.

Collectively, past clinical trials confirmed the preclinical findings that the combination of angiostatic drugs with tumor irradiation could provide opportunities to improve patient outcome. However, the combination therapy was found to be associated with increased toxicity profiles which pointed toward the need to improve dose-scheduling.

## The present progress in combined angiostatic/radiation therapy

The 2012 paper by Kleibeuker et al. listed 78 ongoing clinical trials combining angiostatic drugs with radiotherapy [24] which was illustrative of the expectations regarding the clinical benefit of this combination treatment strategy. At present, 45 of these trials have been completed and 19 trials are still ongoing. In addition, four have been terminated due to either an insufficient number of participants, unacceptable toxicity or withdrawal of support from the sponsor, whereas the remaining studies did not have a recent status update (Table 2). Out of the 45 completed trials, 22 published study results in PubMed-indexed journals. Of note, the majority of these published trials, i.e., 18, evaluated the combination of (chemo)radiotherapy with bevacizumab (Table 3). Since we recently discussed the opportunities of combining radiotherapy with another widely used angiogenesis inhibitor, i.e., sunitinib [63], we will focus here mainly on bevacizumab.

**Table 2:** Updated list of ongoing clinical trials in 2012

Trial	Phase	Disease^a^	Scheduling^b^	Radiotherapy regime^c^	Chemotherapy	Status^d^	Remark/publication
Bevacizumab							
NCT00570531	II	EC (locoregional)	Conc	25 × xGy	Paclitaxel/cisplatin/5-FU	4	Insufficient number of participants
NCT00354679	II	ES (LA)	Neo/conc	30 × xGy	Cisplatin/irinotecan	1	Results, unpublished
NCT01332929	I	Brain metastases	Neo/conc	15 × 2 or 10 × 3 Gy		1	Levy et al. [109]
NCT00817284	II	GBM	Neo/conc	30 × 2 Gy	Temozolomide vs irinotecan	1	No results/publications
NCT00805961	II	GBM (first-line treatment)	Conc/adj	30 × 2 Gy	Temozolomide/everolimus	1	Hainsworth et al. [77]
NCT00590681	II	GBM (ND)	Adj	30–33 × 1.8–2.0 Gy	Temozolomide	1	No results/publications
NCT01186406	II	GBM (ND)	Conc/adj	30 × 2 Gy	Gliadel/temozolomide	2	Results, no publications
NCT00884741	III	GBM (ND)	Conc/adj	30 × xGy	Temozolomide	1	Gilbert et al. [65]
NCT00943826	III	GBM (ND)	Neo/conc	30 × 2 Gy	Temozolomide	1	Chinot et al. [64]
NCT01102595	II	GBM (unresectable)	Neo/conc	30 × 2 Gy	Temozolomide	1	No results/publications
NCT01022918	II	GBM (unresectable)	Neo/adj	30 × 2 Gy	Temozolomide/irinotecan	1	Chauffert et al. [72]
NCT00597402	II	GBM and gliosarcomas	Conc/adj	33 × xGy	Temozolomide/irinotecan	1	Results, no publications
NCT01209442	II	GBM	Conc/adj	60 Gy in 2 weeks	Temozolomide	2	No results/publications
NCT01013285	II	GBM or gliosarcoma (ND)	Conc/adj	30 × xGy	Temozolomide	2	No results/publications
NCT01443676	II	GBM (eldery patient)	Conc	Unknown		1	No results/publications
NCT00369122	II	Cervical cancer (LA)	Conc	45 Gy in 25 fractions	Cisplatin	1	Results, unpublished
NCT00545792	II	Gynecological cancer (recurrent)	Conc	45 Gy in 25 fractions		1	Viswanathan et al. [78]
NCT00703976	II	HNSCC (LA)	Conc	35 × 2 Gy	Cetuximab/pemetrexed	1	No results/publications
NCT00281840	II	HNSCC (stage III/IV)	Conc/adj	40 × 1.8 Gy	Docetaxel	1	Yao et al. [76]
NCT01004874	II	Malignant glioma (grade IV)	Conc/adj	Standard Rth for 6.5 weeks	Temozolomide/topotecan	2	Results, no publications
NCT00782756	II	Malignant glioma (ND)	Neo/conc/adj	3 × xGy/week for 2 weeks	Temozolomide	2	No results/publications
NCT01478321	II	High-grade recurrent malignant glioma	Conc/adj	25 × xGy	Temozolomide	3	No results/publications
NCT00387374	II	NCSLC (stage IIIB/IV unresectable)	Adj	10 × xGy	Carboplatin/paclitaxel	1	No results/publications
NCT00896181	II	NPC (advanced)	Neo/conc	30–35 × xGy	Doxatel/cisplatin/5-FU/carboplatin	3	No results/publications
NCT00408694	II	NPC (stage IIB–VB)	Conc/adj	33 × 2.12 Gy	Cisplatin/5-FU	1	Lee et al. [74]
NCT00334815	I/II	NSCLC (stage III irresectable)	Conc	35 × xGy	Cisplatin/etoposide	2	Results, unpublished
NCT00402883	II	NSCLC (stage III)	Conc/adj	35 × 1.8 Gy	Pemetrexed/carboplatin	4	Toxicity
NCT00578149	II	NSCLC (stage III)	Conc/adj	35 × xGy	Paclitaxel/carboplatin	1	No results/publications
NCT00307723	I/II	Pancreatic cancer	Conc	35 × xGy	Oxaliplatin/5-FU	4	Insufficient number of patients
NCT00336648	II	Pancreatic cancer	Conc/adj	28 × 1.8 Gy	Gemcitabine	1	No results/publications
NCT00460174	II	Pancreatic cancer (localized)	Neo	15 × 2.4 Gy	Gemcitabine	1	Rezai et al. [110]
NCT00557492	II	Pancreatic cancer (potentially resectable)	Conc/adj	10 × 3 Gy	Gemcitabine	2	Results, unpublished
NCT00349557	II	Prostate cancer (high risk)	Conc/adj	IMRT	Bicalutamide	1	No results/publications
NCT00321685	II	RC (LA non-metastatic)	Conc	28 × 1.8 Gy	Capecitabine/oxaliplatin (FOLFOX)	1	Landry et al. [73]
NCT00113230	II	RC (LA)	Conc	28 × 1.8 Gy	Capecitabine	1	Results, unpublished
NCT01434147	II	RC (LA)	Neo	25 × 1.8 Gy	Capecitabine/oxaliplatin	1	No results/publications
NCT00557713	II	RC (LA)	Neo/conc	28 × 1.8 Gy	Oxaliplatin/capecitabine	5	No results/publications
NCT00842686	II	RC (LA)	Neo/conc	28 × 1.8 Gy	Capecitabine	5	No results/publications
NCT00865189	II	RC (LA)	Neo/conc	25 × 1.8 Gy	FOLFOX/5-FU	1	Borg et al. [71]
NCT01043484	II	RC (localized)	Conc	25 × 1.8 Gy	Capecitabine	1	Salazar et al. [75]
NCT00308516	II	RC (stage II/III)	Conc/adj	28 × 1.8 Gy	FU/FOLFOX6	1	Spigel et al. [83]
NCT01481545	II	RC (poor risk)	Conc/adj	25 × 1.8 Gy		2	No results/publications
NCT00356031	II	Sarcoma	Neo	30 × xGy		5	No results/publications
NCT00308529	II	SCLC (LA)	Neo/conc/adj	34 × 1.8 Gy	Irinotecan/carboplatin	1	Spigel et al. [111]
NCT00387699	II	SCLC (limited stage)	Conc/adj	15 × twice daily xGy	Cisplatin/etoposide	1	No results/publications
Bevacizumab/erlotinib							
NCT00393068	II	EC (operable)	Conc	25 × 1.8 Gy	5-FU/paclitaxel/carboplatin	1	Bendell et al. [70]
NCT00720356	II	GBM or gliosarcoma (ND)	Conc/adj	30 × xGy	Temozolomide	2	No results/publications
NCT00140556	I	HNSCC/NPC	Conc	70 Gy in twice daily 1.25 Gy	Cisplatin	1	Yoo et al. [87]
NCT00392704	II	HNSCC (LA)	Neo/conc	38 × 1.8 Gy	Paclitaxel/carboplatin/5-FU	1	Hainsworth et al. [112]
NCT00280150	I/II	NSCLC (stage III)	Conc	74 Gy in 35 fractions	Carboplatin	1	No results/publications
NCT00614653	I	Pancreatic cancer	Conc	28 × 1.8 Gy	Capecitabine	1	No results/publications
NCT00735306	I/II	Pancreatic cancer	Conc	28 × 1.8 Gy	Raltitrexed/oxaliplatin/5-FU	1	Results, unpublished
NCT00307736	I/II	RC (LA)	Conc	28 × 1.8 Gy	5-FU	1	Blaszkowsky et al. [113]
NCT00543842	I/II	RC (LA)	Conc	28 × 1.8 Gy	Capecitabine	1	No results/publications
Bevacizumab/cetuximab							
NCT00703976	II	HNSCC (LA)	Conc/adj	35 × 2 Gy	Pemetrexed	1	No results/publications
NCT01262859	II	HNSCC (LA)	Neo/con	35–37 × 2 Gy	Cisplatin	4	No results/publications
NCT00968435	II	HNSCC (stage III/IV)	Neo/conc	70 Gy in 33 fractions	Cisplatin	1	No results/publications
Endostar/endostatin							
NCT01158144	II	NSCLC (LA)	Conc	30–33 × 2 Gy	Paclitaxel/carboplatin	1	Sun et al. [104]
NCT01218594	II	NSCLC (LA)	Neo/conc/adj	30–33 × 2 Gy	Doxatel/cisplatin	1	Bao et al. [102]
NCT01211002	IV	NSCLC (LA)	Conc	30–33 × 2 Gy	Etoposide/cisplatin	5	No results/publications
Sorafenib							
NCT00822848	I	Soft tissue sarcoma	Neo/conc	28 Gy in eight fractions	Epirubicin/Ifosfamide	2	No results/publications
NCT00610246	I	Cancer (not eligible for curative treatment)	Neo/conc/adj	X × xGy		1	No results/publications
NCT00544817	II	GBM (postsurgical)	Adj	30 × 2.0 Gy	Temozolomide	1	Hainsworth et al. [92]
NCT00892658	I	HCC	Neo/conc/adj	Three fractions in 2 weeks		2	No results/publications
NCT01328223	II	HCC (advanced)	Conc/adj	23–24 × 2.0–2.5 Gy		5	No results/publications
NCT00892424	I/II	Liver metastasis (unresectable)	Neo/conc	3 × xGy/week for 2 weeks		2	No results/publications
NCT00609934	I/II	RCC with bone metastasis	Conc/adj	10 × 3 Gy		1	No results/publications
SU5416							
NCT00023725	I/II	Soft tissue sarcoma	Neo/conc/adj	25 × xGy		1	No results/publications
NCT00023738	I/II	Soft tissue sarcoma	Conc/adj	2 cycles of 11 × xGy	Doxorubicin/ifosfamide/dacarbazine	1	No results/publications
NCT01308034	I	Non-GIST Sarcomas	Conc	30 × xGy		2	No results/publications
Sunitinib							
NCT01100177	II	GBM (ND)	Neo/conc	30 × 2 Gy		1	No results/publications
NCT01498835	I	Soft tissue sarcoma	Conc	28 x 1.8 Gy		1	Jakob et al. [96, 114]
NCT00753727	I/II	Soft tissue sarcoma	Neo/conc	28 × 1.8 Gy		5	No results/publications
NCT00631527	I	Prostate cancer	Conc	40 fractions of xGy	Hormone therapy	1	No results/publications
Thalidomide							
NCT00049361	II	Brain metastases (ND)	Conc/adj	15 × xGy	Temozolomide	1	No results/publications
NCT00033254	III	Brain metastases	Conc/adj	15 × 2.5 Gy		1	No results/publications
Vandetanib							
NCT00745732	I/II	NSCLC	Neo/conc	15 × 3 or 33–35 × 2 Gy		4	Sponsor withdrew support

^a^Disease: *RCC* renal cell carcinoma, *LA* locally advanced, *HNSCC* head and neck squamous cell cancer, *NSCLC* non-small cell lung cancer, *EC* esophageal cancer, *RC* rectal cancer, *ND* newly diagnosed, *GBM* glioblastoma, *NPC* nasopharyngeal cancer, *HCC* hepatocellular carcinoma, *SCLC* small cell lung cancer

^b^Scheduling: scheduling of angiostatic drug to radiotherapy *neo* neoadjuvant, *conc* concurrent, *adj* adjuvant

^c^Radiotherapy: Rth is applied at a frequency of 5 days/week unless indicated otherwise. When the dose applied is unknown, this is indicated with × Gy

^d^Status: 1 = completed; 2 = active, not recruiting; 3 = recruiting; 4 = terminated/withdrawn; 5 = unknown

**Table 3:** Overview of trials combining RTx with bevacizumab published between 2012 and 2017

Trial	Phase	Disease^a^	Scheduling^b^	Radiotherapy regime^c^	Chemotherapy	Treatment benefit	References
NCT01332929	I	Brain metastases	Neo/conc	15 × 2 or 10 × 3 Gy		1/3 versus 11/15^a^	[109]
NCT00805961	II	GBM (first-line treatment)	Conc/adj	30 × 2 Gy	Temozolomide/everolimus	Yes (PFS)^b^	[77]
NCT01186406	II	GBM (ND)	Conc/adj	30 × 2 Gy	Temozolomide	No (OS and PFS)	[65]
NCT00943826	III	GBM (ND)	Neo/conc	30 × 2 Gy	Temozolomide	Yes (PFS), No (OS)	[64]
NCT01022918	II	GBM (unresectable)	Neo/adj	30 × 2 Gy	Temozolomide/irinotecan	No (PFS)	[72]
NCT00545792	II	Gynecological cancer (recurrent)	Conc	45 Gy in 25 fractions		Yes (PFS)	[78]
NCT00281840	II	HNSCC (stage III/IV)	Conc/adj	40 × 1.8 Gy	Docetaxel	No (PFS)^b^	[76]
NCT00408694	II	NPC (stage IIB–VB)	Conc/adj	33 × 2.12 Gy	Cisplatin/5-FU	No (PFS)^b^	[74]
NCT00460174	II	Pancreatic cancer (localized)	Neo	15 × 2.4 Gy	Gemcitabine	NA^c^	[110]
NCT00321685	II	RC (LA non-metastatic)	Conc	28 × 1.8 Gy	Capecitabine/oxaliplatin (FOLFOX)	No (pCR)^b^	[73, 115]
NCT00865189	II	RC (LA)	Neo/conc	25 × 1.8 Gy	FOLFOX/5-FU	No (pCR)^b^	[71]
NCT01043484	II	RC (localized)	Conc	25 × 1.8 Gy	Capecitabine	No (pCR)	[75]
NCT00308516	II	RC (stage II/III)	Conc/adj	28 × 1.8 Gy	FU (conc)/FOLFOX6 (adj)	Yes (pCR; DFS)^b^	[83]
NCT00307736	I/II	RC (LA)	Conc	28 × 1.8 Gy	5-FU/erlotinib	Yes (pCR)^b^	[113]
NCT00308529	II	SCLC (LA)	Neo/conc/adj	34 × 1.8 Gy	Irinotecan/carboplatin	NA^d^	[111]
NCT00393068	II	EC (operable)	Conc	25 × 1.8 Gy	5-FU/paclitaxel/carboplatin/erlotinib	No (pCR)	[70]
NCT00140556	I	HNSCC/NPC	Conc	70 Gy in twice daily 1.25 Gy	Cisplatin/erlotinib	Yes (OS)^b^	[87]
NCT00392704	II	HNSCC (LA)	Neo/conc	38 × 1.8 Gy	Paclitaxel/carboplatin/5-FU (neo); Paclitaxel/erlotinib (conc)	Yes (PFS)^b^	[112]

^a^Responders according RECIST in patients treated with increasing dose bevacizumab

^b^Compared to historical studies, *NA* not assessed

^c^Study was set up to compare different response measures

^d^Due to early trial closure related to toxicity

### Combining bevacizumab with (chemo)radiotherapy

In 2014, Gilbert et al. and Chinot et al. published the results of two phase III trials that evaluated whether bevacizumab improves the efficacy of standard chemoradiotherapy for patients with newly diagnosed glioblastoma [64, 65]. Both studies were initiated based on previous observations in phase I/II trials that suggested a potential benefit of this combination treatment [66–69]. In the trial of Gilbert et al., bevacizumab (or placebo) was added in the fourth treatment week of concurrent radiotherapy (30 × 2 Gy) plus temozolomide, whereas Chinot et al. started bevacizumab (or placebo) already in the first treatment week of radiotherapy (30 × 2 Gy) plus temozolomide. Gilbert et al. found no benefit of bevacizumab in terms of overall survival (OS; 15.7 vs. 16.1 months; HR 1.13) and progression-free survival (PFS; 10.7 vs. 7.3 months; HR 0.79), but reported a worse quality of life and a decline in neurocognitive function in the bevacizumab group. Patients treated with bevacizumab experienced grade 3 or higher adverse events more frequently [65]. Chinot et al. did observe a prolonged PFS in the bevacizumab group as compared to placebo (10.6 vs. 6.2 months), but also failed to show a significant difference in OS. Again, grade 3 or higher adverse events were more common in the bevacizumab cohort (66.8 vs. 51.3% in the placebo group) [64]. Of note, since the statistical design of the two trials was not comparable, a direct comparison cannot be made. However, both studies point toward a favorable PFS with the addition of bevacizumab to radiotherapy plus temozolomide in newly diagnosed glioblastoma, especially when combination treatment is initiated at the start of radiotherapy. Unfortunately, the favorable PFS is accompanied with increased toxicity. Thus, it remains to be seen if bevacizumab is really a beneficial addition to the first-line treatment in glioblastoma patients.

Overall, the clinical value of combining bevacizumab with radiotherapy is open for debate, especially since most current trials either report no clinical benefit [70–76] or only a (minor) benefit in PFS or pCR (pathological complete response) [77, 78]. For example, several phase II studies have been performed in rectal cancer patients based on promising results in phase I trials [79, 80]. Borg et al. [71] added bevacizumab to neoadjuvant 5-FU and RT for 46 patients with stage III rectal cancer before total mesorectal excision, evaluating the proportion of patients achieving a pathological complete response (pCR; ypT0N0). The study did not reach a significant difference from expected pCR (10.0%) with a rate of 11.4%. Salazar et al. also failed to show a significant difference in pCR with the addition of bevacizumab to capecitabine-based neoadjuvant chemoradiotherapy (CRT) in 44 patients with stage II/III rectal cancer as compared to 46 patients undergoing only CRT (16 vs. 11%, *p* = 0.54) [75]. Comparable observations were reported by Dellas et al. [81]. More recently, Landry et al. reported on the 5-year clinical outcomes of a phase II trial in patients with locally advanced rectal cancer that received preoperative chemoradiation with bevacizumab followed by postoperative chemotherapy (FOLFOX) plus bevacizumab. Despite excellent 5-year OS and DFS, the primary endpoint (30% pCR) was not reached. Moreover, the treatment schedule was associated with substantial neoadjuvant and surgical toxicity. This resulted in low compliance to adjuvant treatment, and therefore, it was recommended to not further explore this combination treatment [73]. Also Kennecke et al. [82], who evaluated preoperative bevacizumab treatment added to oxaliplatin, capecitabine and radiation in 42 locally advanced or low rectal cancer patients, suggested that the results of their study did not justify a phase III trial aimed at exploring the benefit of neoadjuvant bevacizumab in rectal cancer. Of note, Spigel et al. [83] observed an improved pCR rate of 29% in patients with stage II/III rectal cancer who were treated prior to surgery with 5-fluorouracil, bevacizumab and radiotherapy. Also Xiao et al. [84] reported that sandwich-like neoadjuvant therapy with bevacizumab was safe and effective for locally advanced rectal cancer. Possibly, differences in the timing of surgery, i.e., 2–8 versus 6–8 weeks following chemoradiation could underlie the different observations [85]. This should be taken into account when further optimizing dose-scheduling of both treatments in rectal cancer. Regarding scheduling, the patient accrual in a single-arm phase II study by Resch et al. [86] in rectal cancer patients that received bevacizumab concurrent with chemoradiation was terminated due to toxicity. Based on these current results, it can be argued whether the combination of radiotherapy with bevacizumab will provide a clinical benefit to rectal cancer patients.

A similar conclusion can be claimed regarding the combination of radiotherapy and bevacizumab in nasopharyngeal cancer. In a phase II clinical trial by Lee et al. [74], 44 patients with stage IIB–IVB nasopharyngeal cancer received radiotherapy (33 × 1.2 Gy) in combination with three cycles of bevacizumab and cisplatin, followed by standard adjuvant treatment consisting of fluorouracil in combination with bevacizumab. While the study was designed to test toxicity, the estimated PFS was lower than previously reported in standard therapy (75 vs. 86%). The estimated PFS was also not reached in the study by Yao et al. in patients with locally advanced squamous cell carcinoma of the head and neck that received bevacizumab concurrent with chemoradiation followed by maintenance bevacizumab treatment. Nevertheless, they concluded that the regimen could be further studied in appropriately selected patients, especially those not eligible for cisplatin administration [76]. Of note, a study combining both bevacizumab and erlotinib with concurrent chemoradiation in head and neck cancer patients appeared to give favorable locoregional control and OS compared to historical controls. It was suggested to further study this in a randomized study [87].

Altogether, most current studies combining bevacizumab with radiotherapy only show moderate to no clinical benefit at all. In addition, in most studies the combination treatment is associated with increased albeit manageable toxicities. All this is in line with previous observations [24].

### Combining sorafenib with (chemo)radiotherapy

As mentioned previously, most trials on combining angiostatic drugs with irradiation that were published between 2012 and 2015 involved the addition of bevacizumab to radiotherapy. However, a few trials assessed the combination with other angiostatic drugs. For example, several studies evaluated the addition of the tyrosine kinase inhibitor sorafenib to radiation therapy. Brade et al. [88] tested the safety of combining sorafenib with SBRT (up to 51 Gy) in liver cancer patients. They observed a high rate of adverse events and DLTs, predominantly in patients in which a high volume of liver was irradiated. This is in line with reports from Goody et al. and Dawson et al. [89, 90]. Based on these observations, it appears not advisable to combine concurrent sorafenib with SBRT in patients with locally advanced HCC [88]. Interestingly, Chen et al. [91] observed more acceptable toxicities when combining concurrent sorafenib with conventional fractionated radiotherapy (2.0–2.5 Gy/fraction up to 60 Gy). Since the response rate appeared similar compared to historical studies of radiotherapy alone, it was concluded that the schedule could be further investigated, albeit with caution [91]. A comparable conclusion was drawn by Hainsworth et al. [92] who applied maintenance sorafenib plus temozolomide treatment following fractionated radiotherapy (30 × 2 Gy) plus temozolomide in newly diagnosed glioblastoma patients. Of note, the necessity to combine radiotherapy with sorafenib with caution was further exemplified by the observation that sorafenib prior to radiotherapy can result in reduced liver volumes which might require adjustment of the radiation dose [93]. In addition, sorafenib treatment was associated with gastrointestinal perforation after radiotherapy in advanced renal cell carcinoma patients [94]. These findings again illustrate the necessity to gain more insight in the effects of alternative dose-scheduling regimes when combining radiotherapy with angiostatic drugs.

### Combining sunitinib with (chemo)radiotherapy

The opportunities and challenges of this combination treatment were previously reviewed by Kleibeuker et al. [63]. Here, we briefly discuss some of the latest insights. Recently, Jakob et al. published the results of two phase I trials that explored the feasibility of concurrent sunitinib and radiotherapy for treatment of locally advanced soft tissue sarcoma (STS) prior to surgery [95, 96]. They observed acceptable toxicity which was comparable to other studies that evaluated the combination of radiotherapy with angiostatic drugs in STS, including pazopanib [97] and bevacizumab [98]. Based on favorable responses, all these studies recommended further investigation of the combination treatment in future trials [95–98]. However, a single phase Ib/II study of sunitinib with radiotherapy in soft tissue sarcoma reported unacceptable toxicities as well as increased local relapse rates [99]. Interestingly, the initial dose of sunitinib used in this study was higher compared to the other studies (50 vs. 25–37.5 mg), which might explain the observed increased toxicity. Horgan et al. explored the feasibility, tolerability and efficacy of sunitinib adjuvant to surgery in locally advanced esophageal cancer patients that received neoadjuvant chemoradiation (irinotecan/cisplatin + 25 × 2 Gy). This regime appeared feasible although it was poorly tolerated. In addition, the treatment did not show any clinical benefit compared to (historical) controls [100]. All these findings support the previous conclusions by Kleibeuker et al. [63] that effective combination of radiotherapy with sunitinib relies on better insights in the optimal dosing and scheduling of the combination treatment.

### Combining other angiostatic drugs with (chemo)radiotherapy

Besides the trials discussed above, a handful of studies described the combination of additional angiostatic drugs with radiotherapy. For some of these, e.g., SU5416 (semaxanib) and vandetanib, no trial results have been published, possibly because the combination treatment is no longer of interest. For other inhibitors, some information is available. As mentioned above, pazopanib was combined with radiotherapy in soft tissue sarcoma patients [97]. Based on a dose–escalation study, Haas et al. [97] concluded that neoadjuvant pazopanib (daily dose of 800 mg daily for 6 weeks) in combination with 50 Gy (25 × 2 Gy) appeared safe, albeit that toxicity should be carefully monitored in future studies. Of note, a case study reported complete remission of gastric and esophageal metastases in a renal cancer patient after treatment with radiotherapy (10 × 3 Gy) and neoadjuvant as well as adjuvant pazopanib [101]. The study by Haas et al. also reported favorable responses which warrants further studies on the clinical benefit of radiotherapy combined with pazopanib.

Two recent studies evaluated the combination of endostar/endostatin with radiotherapy in NSCLC patients. In the study by Bao et al. [102], patients with unresectable stage III NSCLC were treated with endostar combined with concurrent chemoradiation (docetaxel/cisplatin + 30–33 × 2 Gy). They reported promising short-term efficacy and local control rates, and the treatment regimen was generally well tolerated [102]. These findings are in agreement with a previous study in NSCLC patients and warrant future evaluation of this treatment [103]. On the other hand, Sun et al. evaluated the addition of endostatin to concurrent chemoradiation (carboplatin/paclitaxel + 30–33 × 2 Gy) followed by maintenance chemotherapy + endostatin in patients with unresectable stage III NSCLC. This study was closed early because of unacceptable toxicity, i.e., four out of ten patients presented with grade III pulmonary toxicity [104].

Collectively, the results of the current studies that evaluate the feasibility to combine angiostatic drugs with radiotherapy have not provided remarkable novel insights regarding this treatment regimen. The occurrence of toxicities remains an issue of concern [105]. Combining radiotherapy with bevacizumab generally shows limited efficacy while the combination with other angiostatic drugs has shown favorable responses, but still awaits further confirmation in (randomized) clinical studies. Optimization of dosing and scheduling of both treatment modalities remains one of the key future challenges.

## The future of combined angiostatic/radiation therapy

Based on preclinical studies as well as on different clinical observations, it still appears feasible that the combination of angiostatic drugs with radiotherapy can be a valuable addition to current therapeutic strategies for cancer patients. At the same time, the insights from past and present clinical trials have made it clear that successful clinical implementation of this combination treatment requires considerable investigations. As evident from our current and previous review, there are a large number of studies ongoing that will help to resolve some of the outstanding questions, especially with regard to feasibility and toxicity of combining different angiostatic drugs with different radiation regimes in a broad spectrum of cancer types. Moreover, several novel trials have been initiated in the past 5 years, for example with bevacizumab (Table 4). The results of all these trials will help to make the necessary steps to bring effective combination therapy to cancer patients.

**Table 4:** Newly initiated trials combining bevacizumab with radiotherapy (2012–2017)

Trial	Phase	Disease^a^	Scheduling^b^	Radiotherapy regime^c^	Chemotherapy	Status^d^
NCT01730950	II	GBM	Conc	IMRT, 3D-CRT, or proton beam RT 5 days a week for 2 weeks	None	2
NCT01746238	I	STS	Conc	6 weeks, 5 days a week	Doxorubicin	3
NCT02313272	I	GBM	Conc	Hypofractionated SRT	Pembrolizumab	3
NCT01871363	II	RC	Conc	25 × 2 Gy	Capecitabine	5
NCT01743950	II	GBM	Conc	27 × 2 Gy (PRDR)	None	3
NCT01569984	II	mCRC	Neo	Up to 60 Gy in six fractions, alternating weekdays for 2 weeks	None	1
NCT02185352	II	BM in BC	Neo	WBRT	Etoposide, cisplatin	3
NCT01580969	Ib/II	Glioma	Conc	Individually determined	Minocycline	3
NCT01588431	II	HNSCC	Neo/conc	5 weeks, 70 Gy	Docetaxel, cetuximab, cisplatin	2
NCT01818973	II	RC	Neo/conc	5 weeks, 50 Gy	Capecitabine + oxaliplatin	3
NCT01554059	II	RC	Neo/conc	5 weeks, 50 Gy	5-FU, oxaliplatin	1
NCT02812641	II	EC	Conc	4 weeks, 40 Gy	Cisplatin, 5-FU	3
NCT02672995	I	BM	Conc	Three fractions, 18–27 Gy	None	3

^a^Disease: *GBM* glioblastoma, *STS* soft tissue sarcoma, *RC* rectal cancer, *mCRC* metastatic colorectal cancer, *BM* brain metastasis, *BC* breast cancer, *HNSCC* head and neck squamous cell cancer, *EC* esophageal cancer

^b^Scheduling: scheduling of angiostatic drug to radiotherapy *neo* neoadjuvant, *conc* concurrent, *adj* adjuvant

^c^Radiotherapy: radiation is applied at a frequency of 5 days/week unless indicated otherwise. When the dose applied is unknown, this is indicated with × Gy

^d^Status: 1 = completed; 2 = active, not recruiting; 3 = recruiting; 4 = terminated/withdrawn; 5 = unknown

One of the most urgent issues to address involves the optimal dose-scheduling of both treatment modalities, not only to improve treatment efficacy, but also because the results from past studies indicated that inadequate dose-scheduling can induce severe toxicities [24, 63]. With regard to dosing, it is important to explore how alterations in dosing of either treatment affect the toxicity and efficacy of combination therapy. For example, Carlson et al. and Omura et al. explored the addition of bevacizumab to hypofractionated radiotherapy in newly diagnosed glioblastoma patients [106, 107]. In the latter study, patients received hypofractionated stereotactic radiotherapy (6 × 6 + 4 Gy over 2 weeks) with concurrent and adjuvant temozolomide plus bevacizumab. The regime was identified as safe and was found to have a comparable effect on OS as compared to historical standard treatment. Moreover, the reduced treatment period appears more convenient for cancer patients [106]. The study by Carlson et al. [107] also reported that the addition of concurrent/adjuvant bevacizumab to hyperfractionated IMRT (10 × 6 Gy) did not improve OS while a somewhat higher rate of grade 3 toxicity was observed. Nevertheless, these studies provide the first evidence that altered dose-scheduling can be explored in order to improve treatment efficacy. In this light, our recent preclinical results are also of interest. We observed that concurrent combination treatment allowed a 50% reduction in dosing of the angiostatic drug sunitinib without affecting the therapeutic efficacy of conventional fractionated radiotherapy [23, 28]. Of note, the low dose was also effective in combination with single high-dose irradiation. These observations could provide opportunities to improve combination treatment both in the curative and in the palliative setting. However, the applicability of such approaches in a clinical setting still awaits confirmation.

Apart from dosing, the scheduling of both treatments is also of importance. Different effects have been observed between concurrent and (neo)adjuvant combination of angiostatic drugs with radiotherapy [24, 63]. Recently, Avallone et al. [108] reported on differential clinical effects of combining bevacizumab with chemoradiation either concomitantly or sequentially in high-risk locally advanced rectal cancer patients. While the endpoint was reached using the sequential schedule, the concomitant schedule arm was terminated early because of inconsistent activity. Also, toxicity and postoperative complications appeared to be higher after concomitant treatment [108]. This is illustrative of the importance of optimal scheduling, and it is therefore essential that the effect of scheduling is further explored in future studies.

Finally, future studies should aim to integrate insights on tumor perfusion with the observed response to therapy. As described above, the main rationales to combine angiostatic drugs with radiotherapy are (1) to improve tumor perfusion and oxygenation by vessel normalization and (2) to counteract radiation-induced tumor (re)vascularization. Both aims require different approaches with regard to dose-scheduling. Thus, it is vital in obtain information of tumor perfusion and oxygenation prior to treatment planning but also to monitor changes in these parameters during treatment. This could improve treatment efficacy. Several of the trials that were discussed here also included perfusion measurements or explored (bio)markers that could predict response to therapy. However, it is outside the scope of the current review to discuss the insights of these studies regarding these issues. It suffices to state that the development and implementation of noninvasive imaging techniques to measure perfusion and early tumor responses are important to better explain and/or predict the response to the combination of angiostatic drugs with radiotherapy.

## Conclusions

Despite the limited clinical efficacy of angiostatic drugs as monotherapeutics, there is ample evidence that angiostatic therapy can be valuable when combined with other treatment modalities, including radiotherapy. This involves the beneficial effects of angiostatic drugs on tumor perfusion prior to and during radiation as well as their inhibitory effects on tumor (re)vascularization during or after radiation. Past and present clinical trials that combined angiostatic drugs with radiotherapy indeed showed that this approach can improve therapeutic outcome. However, this is mainly observed in phase I/II trials and actual validation of clinical benefit awaits confirmation in larger randomized phase III trials. Moreover, variable efficacy as well as increased toxicity has been reported when angiostatic drugs are combined with radiotherapy. This is most likely due to non-optimal dosing and inadequate scheduling of both treatment regimes. Thus, exploring the close relation between dose-scheduling represents the key challenge for future research regarding combination treatment. This directly relates to the development of rapid and noninvasive imaging strategies in order to measure tumor perfusion prior, during and after treatment. This will help to optimize current approaches to improve treatment strategies and to make effective combination therapy available for cancer patients in daily clinical practice.
